# Characterization and Reliability of Caprylic Acid-Stearyl Alcohol Binary Mixture as Phase Change Material for a Cold Energy Storage System

**DOI:** 10.3390/ma14237418

**Published:** 2021-12-03

**Authors:** Hamza Ayaz, Veerakumar Chinnasamy, Honghyun Cho

**Affiliations:** 1 Graduate School, Chosun University, 309 Pilmundaero, Gwangju 61452, Korea; hamzaayaz@chosun.kr; 2 Department of Mechanical Engineering, Chosun University, 309 Pilmundaero, Gwangju 61452, Korea; veerakumar@chosun.ac.kr

**Keywords:** cold thermal energy storage (CTES), phase change material (PCM), fatty acid, fatty alcohol, thermal reliability, corrosion test

## Abstract

This study reports the in-depth investigation of the thermophysical properties and thermal reliability of caprylic acid-stearyl alcohol (CA-SA) eutectic phase change material (PCM) for cooling applications. The phase diagram of CA-SA showed a eutectic point at a 90:10 molar ratio. The onset melting/freezing temperature and latent heat of fusion of caprylic acid-stearyl alcohol from the differential scanning calorimetry (DSC) were 11.4 °C/11.8 °C and 154.4/150.5 J/g, respectively. The thermal conductivity for the prepared eutectic PCM in the solid phase was 0.267 W/m.K (0 °C), whereas, in the liquid phase, it was 0.165 W/m.K (20 °C). In addition, the maximum relative percentage difference (RPD) marked at the end of 200 thermal cycles was 5.2% for onset melting temperature and 18.9% for phase change enthalpy. The Fourier transform infrared spectroscopy (FT-IR) result shows that the eutectic PCM holds good chemical stability. Corrosion tests showed that caprylic acid-stearyl alcohol could be a potential candidate for cold thermal energy storage applications.

## 1. Introduction

**Figure 1:**
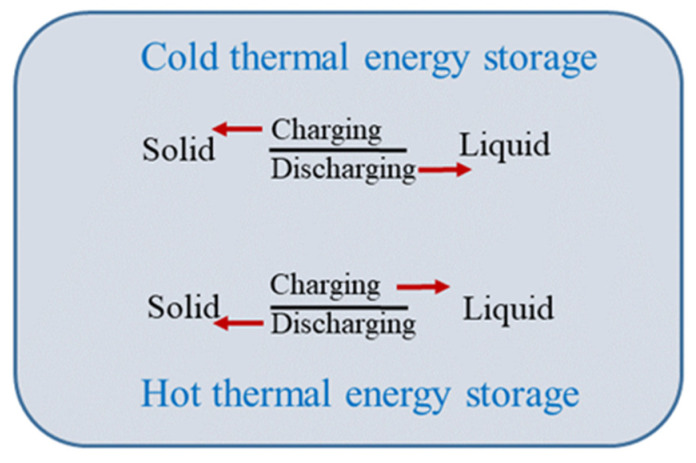
Mechanism of hot and cold thermal energy storage.

The ever-increasing need for energy necessitates innovative approaches to its production and storage. To effectively use ample energy sources available, such as solar energy, a crucial component is required: a proficient and economical heat storage system to compensate for its intermittent nature. Thermal heat storage (TES) is getting more attention due to supply and demand differences, shaving peak load, or enhanced system performance. TES systems store energy during charging when an excess amount of heat is available and retrieve energy during discharging for utilization. TES systems consist of different mechanisms to store heat, namely sensible heat storage, latent heat storage, and thermochemical heat storage. Latent heat thermal energy storage (LHTES) working with phase change material (PCM) is preferred due to high energy density and isothermal energy storage [1,2,3,4]. When a PCM is incorporated into available systems, experimental investigations and numerical simulations significantly improve thermal energy storage capacity. A recent study concluded that a TES system for a solar flat-plate collector with PCM could store 20% more thermal energy than the water storage system [5]. Abdo et al. [6] performed an experiment utilizing bioPCM–M51-Q24 (sheets) with a passive cooling technique for thermal comfort in a building. The result showed chamber temperature with PCM full + windcatcher, PCM full (sheets on walls, ceiling, and floor), PCM sheets on wall and floor, PCM sheet on the wall, and no PCM. The temperature drop in the chamber with PCM (20.5 Kg) was 2.78 °C for a total duration of 5 h, which was a 9.33% drop. LHTES has various applications and was previously utilized for floor heating [7], cold energy recovery in storage plants [8], solar energy storage systems [9], and building cooling applications [10]. Latent heat thermal energy storage is classified as hot or cold thermal energy storage, as shown in Figure 1. Cold or hot thermal energy storage can be defined based on the discharge mechanism for a specific application and not based on the temperature range. For example, the cold thermal energy storage for solid-liquid phase change material depends on the work done during discharge and the phase transition from solid to liquid for CTES. Following this mechanism, latent heat storage can be termed cold thermal energy storage based on application.

At present, the energy stored at low temperature, which is termed cold thermal energy storage (CTES), is gaining more attention. The temperature for CTES with PCM ranges from 30 °C to sub-zero temperature, including air-conditioning assisted PCM, refrigeration system, food processing, and drug transportation. PCMs utilized in these systems help to shift electrical energy demand during peak and off-peak hours. Therefore, CTES with PCM will augment the system performance and give more operational flexibility by shifting the energy load [11,12,13,14]. Based on the chemical composition of PCMs, they are classified as organic (paraffin, non-paraffin) and inorganic (salt hydrates) phase change materials. The organic PCM has potential properties such as congruent phase change, non-toxic nature, lower supercooling, good nucleation rate, and lower cost. Moreover, some organic PCMs, such as as fatty acids, can be prepared from sustainable or reused raw materials like animal fat, palm oil, coconut oil, and animal milk. Fatty acid as a phase change material was studied in detail for thermal energy storage [15].

Kumar et al. [16] investigated stearyl alcohol as a phase change material for passive cooling of a fuel cell to maintain its temperature below 60 °C. The result showed at a power density of 0.30 W/cm^2^ with no cooling, the temperature passed 60 °C, whereas with PCM, the temperature maintained below the specified mark and at 0.35 W/cm^2^, PCM could hold the temperature 70% more compared to no cooling, which depicts the potential of stearyl alcohol for thermal energy storage. Konuklo et al. [17] prepared microcapsules of caprylic acid with wall materials (urea, melamine, and urea and melamine) for thermal energy storage. The detailed investigation confirmed the formation of microcapsules and showed latent heat of fusion for melting/freezing was 93.9/106.1 J/g. Naturally available organic PCMs have fixed phase change temperatures, which limit their applications. A new class of phase change materials was introduced, namely eutectic PCM (binary/ternary mixture), which can be developed with desired phase change temperatures for any specific application. Eutectic PCM is a combination of two or more naturally available PCMs blended with a specific mass proportion of each to have a congruent phase change [18]. The binary mixture is getting a lot of attention as one of a limited number of PCMs for cold thermal energy storage. In the previous studies on the eutectic PCMs, Veerakumar et al. [19] studied the thermophysical characteristics of a new binary combination of fatty acid and a fatty alcohol, namely capric acid–cetyl alcohol with a 70:30 molar ratio for the CTES, as well as their compatibility with other metals. They reported that the peak melting point temperature and freezing point temperature during phase transition were 22.8 °C and 11.97 °C, respectively, with latent heats of fusion during melting (ΔH_m_) and freezing (ΔH_f_) of 144.92 and 145.85 J/g. After 1000 cycles of accelerated thermal cycling, the melting point temperature changed by 6.37%, and the latent heat of fusion dropped by 18.35%. The author also reported that the melting and freezing points were determined to be 18.7 °C and 22.46 °C, respectively, with ΔH_m_ and ΔH_f_ of 151.5 and 151.6 J/g when a binary mixture of lauric acid–myristyl alcohol combined with a 40:60 molar ratio [20]. In addition, the novel binary mixture reported was stable and a good addition to indoor thermal comfort phase change materials. Zuo et al. [21] reported caprylic acid and 1-dodecanol in a 70:30 molar ratio to make an organic binary combination for the CTES system. Their results revealed that a phase transition temperature was 6.52 °C during melting, a degree of supercooling was 2 °C, and latent heat of fusion (ΔH_m_) was 171.06 J/g. The binary mixture’s thermal stability was tested for 60 and 120 melting/freezing cycles, and no significant changes in thermophysical characteristics were found.

For cooling purposes, a binary combination of capric acid and lauric acid was prepared by Dimano et al. [22] with the addition of pentadecane. To attain the eutectic point, the capric acid and lauric acid (CA–LA) combination was initially mixed in a 65:35 molar ratio, and the melting point temperature was determined to be 18 °C with a phase enthalpy of 140.8 J/g. To lower the melting temperature even further, three different combinations of pentadecane with 0.1, 0.3, and 0.5 mole fractions were mixed into a CA–LA mixture and the energy storage performance was evaluated in a shell and tube heat exchanger. Among the various molar fractions of the pentadecane, the CA–LA combination containing a 0.1 mole fraction of pentadecane outperformed the other molar concentrations with a phase transition temperature of 13.2 °C and a phase enthalpy of 142.2 J/g. The melting points of three additions (methyl salicylate, cineole, and eugenol) were 12.2 °C, 12.3 °C, and 13.9 °C, respectively, with latent heats of fusion of 126.7, 111.6, and 117.8 J/g, respectively [23]. Wang et al. [24] prepared a binary combination of caprylic acid-nonanoic acid for cooling purposes with an 81.4:18.6 molar ratio. The DSC method was utilized to measure the latent heat of fusion during melting of 171.06 J/g and a melting point temperature of 7.6 °C. Furthermore, they studied the change in melting point temperature and latent heat of fusion by completing 100 melting/freezing cycles, which revealed no discernible change. Nadiya et al. [25] developed an efficient binary combination of lauryl–cetyl alcohol with an 80:20 molar ratio and examined its efficacy in the refrigeration system chamber. The peak phase transition temperature during melting and freezing was 20.01 °C and 12.04 °C, respectively, with latent heats of fusion of 191.63 and 189.51 J/g. The binary combination maintains the chamber temperature stable for an extended period, making it an excellent fit for the CTES system. Xing et al. [26] investigated the binary mixture of caprylic acid-palmitic acid having a eutectic point at 90:10. The melting phase’s onset/peak phase change temperature was 6.539 °C/11.332 °C, whereas the freezing phase change temperature was 4.312 °C/1.796 °C. The obtained melting and freezing phase enthalpies were 116.477 and 116.235 J/g, respectively.

Guo [27] developed a binary PCM combination of capric acid and dodecanol with a molar ratio of 60:40 for refrigeration applications. The DSC method was initially employed to study the thermal characteristics of the produced binary combination. The phase change temperature was 8.9 °C with a phase change enthalpy of 156.6 J/g. Eutectic PCM for building energy storage at low temperature is utilized by infiltering PCM into the supporting building material. Wang et al. [28] prepared a new eutectic PCM of tetradecanol-lauric acid with the addition of expanded perlite-aluminum for building heat storage. The thermal properties of composite PCM were T_m_ = 24.9 °C with ΔH_m_ of 78.2 J/g and T_f_ = 25.2 °C with ΔH_f_ = 81.3 J/g, respectively. The thermal reliability was analyzed for 2000 thermal cycles, which did not show any significant variation in the thermal properties. Summary of the reported binary eutectic PCM materials (fatty acid-fatty acid, fatty acid-fatty alcohol) for cooling applications are presented in Table 1.

The available phase change materials for air-conditioning and refrigeration applications are limited in number. It is vital to explore more PCMs having melting point temperature in this range. To attain a melting point temperature for any specific application eutectic PCM is the priority that can be prepared with desired phase change temperature. Therefore, this study discusses a novel organic binary PCM combination with a 90:10 molar ratio caprylic acid-stearyl alcohol for the cold energy storage system. Because this binary mixture has not been investigated in previous literature, it is necessary to find out key information prior to utilization for real-time applications. The thermophysical properties were determined, including melting point temperature and phase change enthalpy, and thermal conductivity was investigated in this article. In addition, thermogravimetric analysis and Fourier transform infrared spectroscopy (FT–IR) were used to evaluate thermal and chemical stability. The prepared eutectic PCM was put through 200 continuous thermal melting/freezing cycles to evaluate thermal property reliability. The binary mixture compatibility with various metals utilized in the CTES system’s fabrication was examined by the experimental method. This effort will pave the way for the development of an efficient CTES system for cooling applications in various systems.

## 2. Materials and Methods

### 2.1. Materials

**Figure 2:**
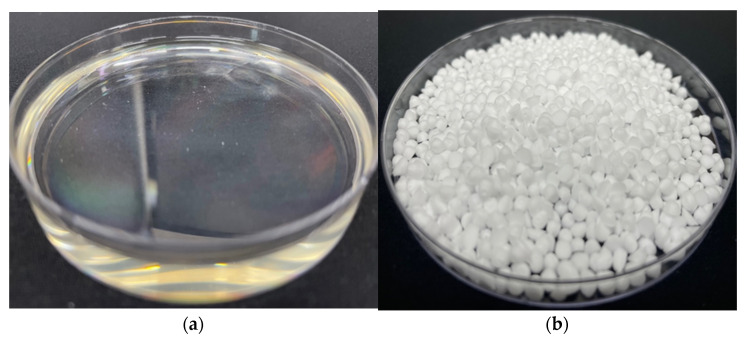
Pure materials at room temperature (25 °C): (**a**) caprylic acid, (**b**) stearyl alcohol.

The pristine materials (caprylic acid and stearyl alcohol) were provided by Daejung Chemicals and Metals, Republic of Korea. Caprylic acid (98%) and stearyl alcohol (99%) are organic phase change materials (PCMs) classified, respectively, as a fatty acid and a fatty alcohol. Figure 2 shows caprylic acid and stearyl alcohol at a room temperature of 25 °C. Caprylic acid is a saturated fatty acid with a pale yellowish color having a pungent smell that remains in a liquid state at room temperature and is found naturally in the milk of mammals. It is chemically represented by C_8_H_16_O_2_ and commercially produced through the oxidation of aldehyde having eight carbons. Stearyl alcohol (C_18_H_38_O) is saturated fatty alcohol that appears as small white granules at temperatures <30 °C. The thermal properties of the pure materials are presented in Table 2.

### 2.2. Preparation of the Binary Mixture Caprylic Acid-Stearyl Alcohol

The eutectic point of the binary mixture has to be deduced in order to analyze the thermal properties. The theoretical estimation with the phase equilibria thermodynamic model (Schröder van Laar equation) noted in Section 3.1 was first used to initiate forming various binary solutions of different mass ratios of the pure materials. The empirical model can predict melting point temperature for the solid-liquid ideal system, which can assist in constructing the phase diagram. The physical properties of the pristine materials are utilized to calculate the predicted liquid line, which can limit the experimental time and cost. The detailed derivation can be found in open literature [29]. Based on the theoretical calculations, a mass proportion of 60–100% of the pure materials was amalgamated with an increment of 10% for each mixture. The pure materials were first weighed with a precision balance (FZ-300iWP, USA) with an accuracy of ±0.001 g to add the accurate mass proportion of each material. The binary mixture was heated to 80 °C and mixed properly with a hot plate and magnetic stirrer (GLHPS-C12, Korea). Finally, the binary mixture was cooled to room temperature.

### 2.3. Thermophysical Properties Determination

The thermal properties of the pure materials and binary mixture prepared were determined with DSC (204 F1 Phoenix, Netzsch, Germany) with an accuracy of <1% for enthalpy. A sample of 5 mg was sealed in an aluminum pan and investigated for the latent heat of fusion (ΔH) and onset/peak melting point temperature (T_onset_ and T_peak_) based on the heat flux principle. The DSC was performed in an inert gas environment. The DSC measurement was performed for three cycles and the average thermal properties are noted. The endothermic and exothermic heat curves were measured at a rate of 10 °C/min increments. The samples were analyzed in the temperature range of −20 °C to 80 °C.

The thermal conductivity of the prepared binary mixture was characterized in the solid and liquid state by the transient hot-wire method using a thermal property analyzer (Decagon KD2 Pro, Pullman, WA, USA) with an accuracy of ±5% (liquid state) and ±10% (solid state). The KD2 Pro analyzer consists of a low-power 16-bit microcontroller for data processing, an analog-to-digital converter, and a probe holding a heater and sensor. The analyzer comes with different needles for liquid and solid states: single-needle (KS-1), which is utilized to measure thermal conductivity in a liquid state, and double-needle (SH-1) for the solid state. The analyzer gives the heat pulse and waits for 30 s to stabilize the temperature and record the temperature difference for the next 30 s by every second to calculate the thermal conductivity. The sensor was set upside down in a 50 mL glass bottle. The measured data were converted with an analog-to-digital converter to display on the screen.

The thermal stability of the binary mixture was investigated by a thermogravimetric analyzer (TGA) (TGA-50, Tokyo, Japan) with an accuracy of ±0.001 mg. Thermogravimetric analyzer reports the change in mass concerning the temperature rise. The sample was placed in an open aluminum pan with a heating rate of 10 °C/min and a temperature range of 25 °C–350 °C. To monitor the decomposition of the sample, an inert environment in the furnace was maintained with argon gas at a flow rate of 50 mL/min. The decomposition temperature of the binary mixture can be deduced from the TGA curve, where a substantial change in the mass can be observed.

### 2.4. Thermal Cycling and Compatibility of Caprylic Acid-Stearyl Alcohol

The accelerated thermal cycling was performed to investigate the thermal and chemical stability of the caprylic acid-stearyl alcohol. The binary mixture prepared was placed into a glass container and subjected to 200 freezing/melting cycles, where the thermal properties and chemical structure were examined at the 0th cycle, 100th cycle, and 200th cycle. A constant thermal bath was utilized in which the temperature was set below its freezing point to solidify the binary mixture completely, followed by a heating cycle with a temperature higher than its melting point. One complete thermal cycle was defined with freezing followed by melting of the caprylic acid-stearyl alcohol completely.

The chemical stability of the binary mixture was analyzed by Fourier-transform infrared spectroscopy (FT-IR) spectrometer (Vertex 70v/Hyperion 2000, Billerica, MA, USA). The infrared spectroscopy was performed in attenuated total reflectance mode. For the preparation of the sample, a few drops were placed on the Kbr pellet, pressed by applying a force of eight tones, and allowed to dry. After preparing the sample, the spectral data were recorded in the range of 4000–450 cm^−1^.

The compatibility of the binary mixture was examined with three different metals utilized for the fabrication of thermal energy storage. Three different metals, namely aluminum, copper, and carbon steel, were selected for the corrosion test of the binary mixture. The standard procedure recommended by American Standard for Testing and Materials (ASTM G1-03) [30] was adopted. First, rectangular metal strips were cut with dimensions 5 × 1 cm height and width, respectively. Next, the metals strips were cleaned with acetone and allowed to dry. After preparing the metal strips, they were weighed with the precision balance before being subjected to the binary mixture. Finally, the metals were placed into a binary mixture and corrosion tests were conducted for a total of 12 weeks. Corrosion rates were calculated for the 1st week, 4th week, and 12th week. The glass container was enclosed to have minimal contact with the surroundings.

## 3. Results and Discussion

### 3.1. Thermal Properties, Thermal and Chemical Stability of the Caprylic Acid-Stearyl Alcohol Binary Mixture

**Figure 3:**
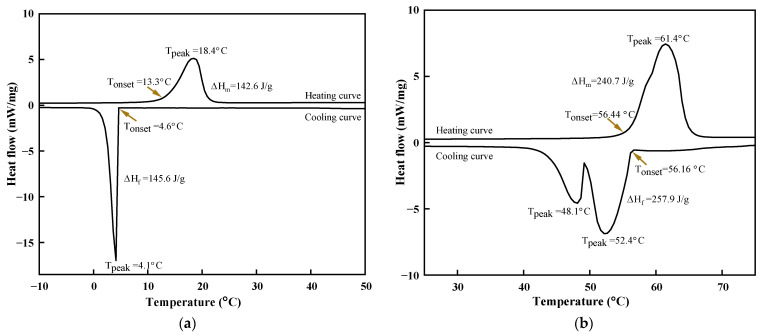
Thermograph of the pristine materials: (**a**) caprylic acid, (**b**) stearyl alcohol.

The thermograph from the DSC analysis represents heat stored/retrieved with the onset/peak melting point temperature and enthalpy change on the fusion of the pure materials is shown in Figure 3a,b. The two thermographs depict the heating and cooling curves of the materials with positive and negative heat flow, showing the phase transition and energy storage/retrieval. The onset/peak melting point temperature and latent heat of fusion for caprylic acid and stearyl alcohol measured were 13.3 °C/18.4 °C and 142.6 J/g, 54.4 °C/61.4 °C and 240 J/g, respectively. The cooling curve represents the onset/peak freezing point temperature with enthalpy change on fusion 4.6 °C/4.1 °C and ΔH_f_ of 145.6 J/g for caprylic acid and56.66 °C/52.4 °C with 257.9 J/g for stearyl alcohol [21,31]. The endotherm of the even number of straight-chain fatty alcohols transforms into three different polymorphs having different thermodynamic stability over the temperature and pressure range. The two peaks in the exothermic curve and a small bump in the endothermic curve of stearyl alcohol can be resolved by peak separation.

In the heating curve, the transition occurs between the ordered phase γ and rotator phase α, the melting points of γ and α are very close, which results in a small shoulder on the heating curve. The two peaks represent the liquid-solid phase and solid-solid transformation α → γ prior to stable crystal shape in the cooling curve. The degree of supercooling is more pronounced in a solid-solid phase transition, whereas from liquid to α is quite low. The supercooling degree is a detrimental factor linked to PCM, which has a negative effect on overall heat storage/retrieval performance. The supercooling degree can be calculated by measuring the difference in the melting and freezing point temperature. The degree of supercooling is attributed to cooling rate, material purity, and operating conditions. The degrees of supercooling for caprylic acid and stearyl alcohol were 8.7 °C and 0.42 °C, respectively.

The eutectic point of the binary mixture was theoretically calculated by the Schröder van Laar equation as shown in Equation (1) for different mass proportions of pristine materials [19].

(1) $$lnX_{A}=\frac{\Delta H_{A}}{R}\left(\frac{1}{T_{A}}-\frac{1}{T}\right)$$

where *X_A_* is the molar mass of the base material, *R* is the general gas constant (8.31 J/mol.K), Δ*H_A_* is the latent heat of fusion (J/g), *T_A_* is the melting point of the base material (K), and *T* is the melting point (K) calculated for different proportion. Using Equation (1), the onset melting point temperature for the caprylic acid/cetyl alcohol binary mixture was calculated and it was 12.7 °C for a 90:10 molar mass ratio, which was less than the melting point of the individual material.

**Figure 4:**
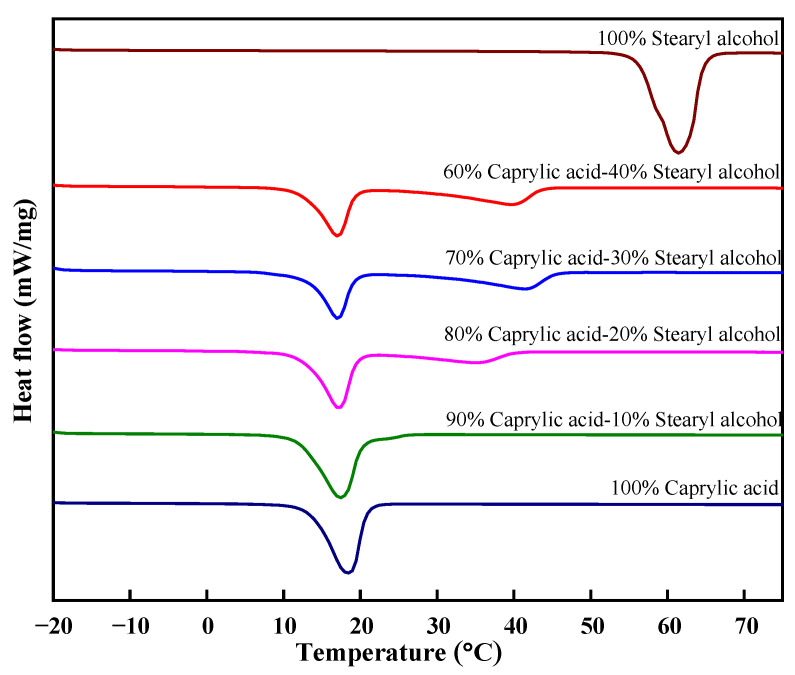
Heating curves of the different mass proportions of a binary mixture.

The DSC analysis was performed for the binary mixture at the theoretical eutectic point and other nearby mass proportions. The heating curve for the mass proportion 60–100% of caprylic acid-stearyl alcohol is graphically presented in Figure 4. It is observed from the endotherm that all the binary mixture proportions have first onset melting point temperatures that are almost fixed, known as the solidus temperature. In contrast, the second melting point temperature (liquid temperature) varies with reducing the mass proportion of stearyl alcohol. The binary mixture having a 90% mass proportion of caprylic acid and 10% stearyl alcohol shows one melting point temperature of 11.4 °C by the DSC analysis, which is similar to the theoretical estimated eutectic point for the prepared binary mixture.

**Figure 5:**
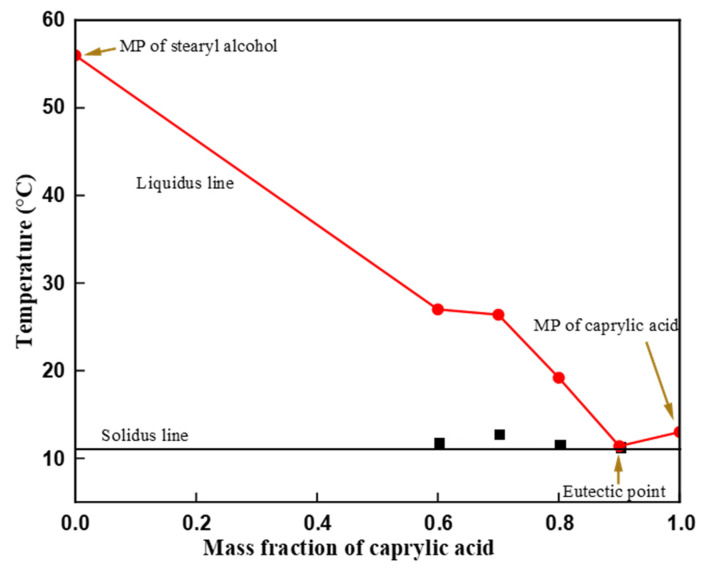
Phase diagram of the caprylic acid-stearyl alcohol.

The phase diagram of the eutectic binary mixture was plotted based on the measured onset melting point temperature with the DSC for different mass proportions. The onset melting point temperature of the binary mixture plotted against the mole fraction of the caprylic acid is shown in Figure 5. The solidus temperature remained approximately invariant whereas the liquidus line varied with the incremental mole fractions of the caprylic acid. The second peak diminished slowly with the increase in mass fraction of the caprylic acid; a minimum onset melting point in the curve was marked at 0.9 and 0.1 mole fraction of caprylic acid and stearyl alcohol, respectively. Thus, the minimum onset melting point of 11.4 °C was confirmed as the eutectic point for the caprylic acid-stearyl alcohol binary mixture.

**Figure 6:**
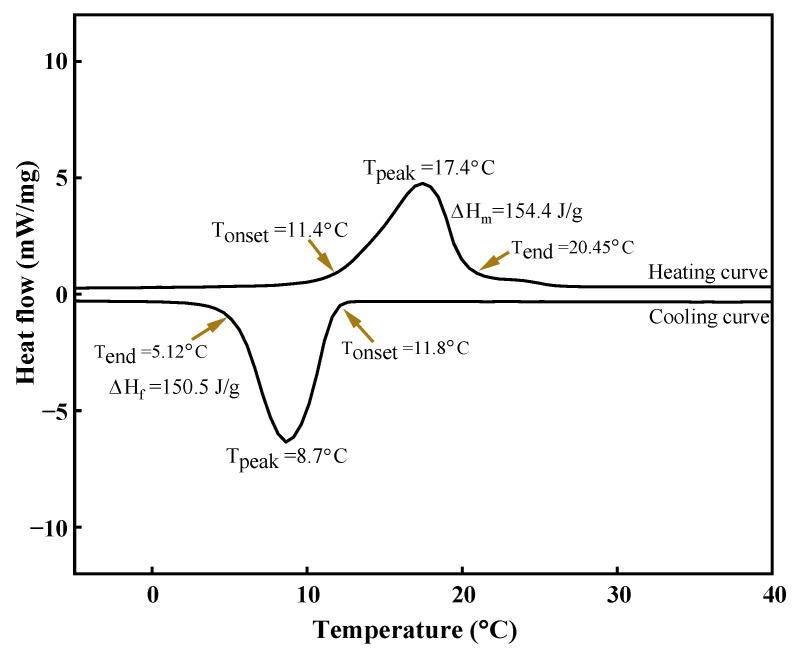
DSC curve of the caprylic acid-stearyl alcohol (CA-SA).

The DSC heating and cooling curve for the eutectic caprylic acid-stearyl alcohol mixture is shown in Figure 6. The endotherm for the binary mixture shows the onset/peak melting point temperature of 11.4 °C/17.4 °C with the latent heat of fusion (Δ*H_m_*) of 154.4 J/g. The exotherm presents an onset/peak freezing point temperature of 11.8 °C/8.7 °C with Δ*H_f_* of 150.5 J/g. The specific heat of the eutectic material was measured with DSC at three different states. Specific heat in solid phase (1.690 J/g.K at 0 °C), during phase change (27.2 J/g.K at 16.9 °C), and in liquid phase (1.9 J/g.K at 26.9 °C). The caprylic-palmitic acid eutectic PCM reported in the literature has an 11.33 °C melting temperature with 116.47 J/g [23]. The eutectic PCM prepared in this work has a higher latent heat of fusion, which makes it a good fit for CTES applications. The degree of supercooling is an undesirable factor linked to the PCM and was higher for the pure materials utilized to prepare the binary mixture. The befitting thermal properties and lower degree of supercooling make the novel eutectic mixture suitable for cooling applications. The onset melting point temperature of the caprylic acid-stearyl alcohol binary mixture makes it a potential candidate for CTES, as few PCMs exist in this temperature range [32].

**Figure 7:**
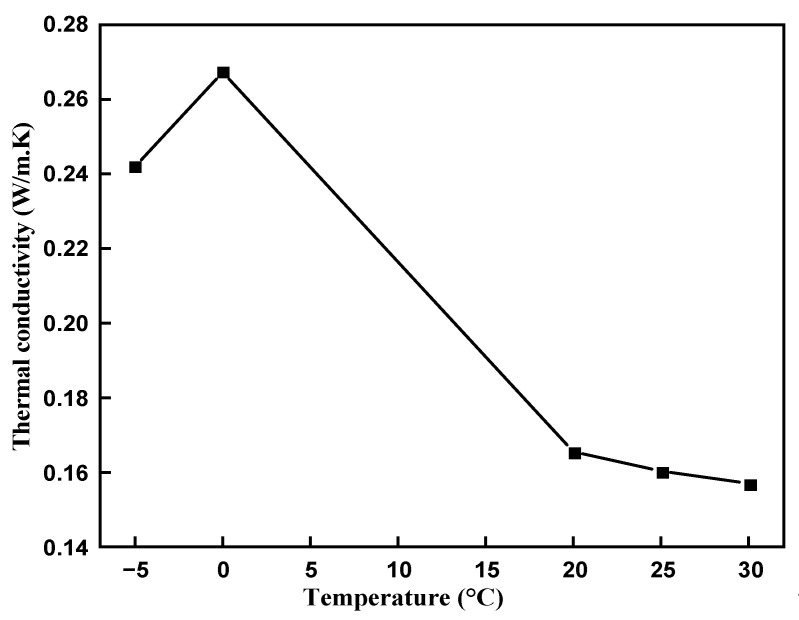
Thermal conductivity of the eutectic PCM at solid and liquid phases.

The thermal conductivity is a significant property of PCM for enhancing the performance of the heat transfer during phase transition. The thermal conductivity of the prepared binary mixture was measured in the solid and liquid phases and it is shown in Figure 7. The organic liquids have a lower thermal conductivity in the range of 0.1–0.2 W/m.K [33]. The measured thermal conductivity in the solid phase was 0.242 and 0.267 W/m.K at −5 °C and 0 °C, whereas in the liquid phase it was 0.165, 0.160, and 0.157 W/m.K at 20 °C, 25 °C, and 30 °C, respectively. The thermal conductivity for each temperature was measured three times and the average value was marked with uncertainty in solid phase ((SH-1) ±10%) and liquid phase ((KS-1) ±5%). The thermal conductivity in the solid phase was more pronounced due to the tightly packed molecules [34]. The measured thermal conductivity for the pure caprylic acid was approximately 0.15 W/m.K, whereas for stearyl alcohol it was 0.248 W/m.K [35,36]. Therefore, the thermal conductivity of the prepared eutectic mixture is in good agreement with pure materials reported in the literature, which will enhance its performance for the cooling application.

**Figure 8:**
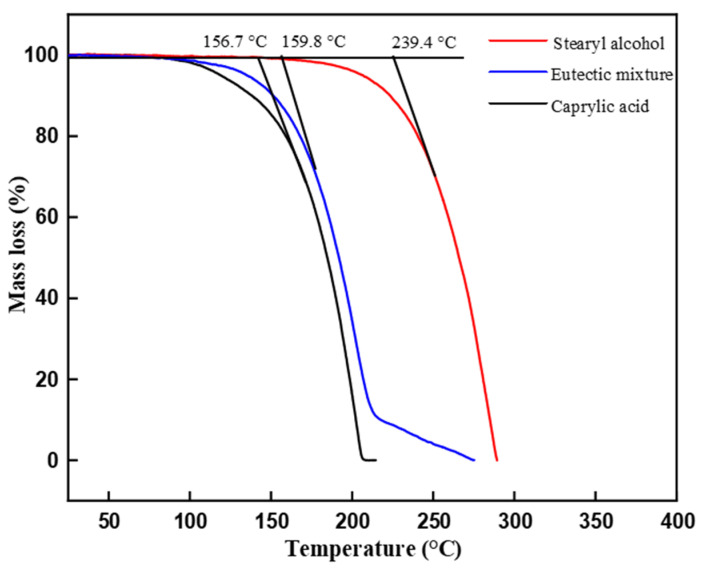
TGA analysis of pure materials and prepared eutectic PCM.

The thermal stability of the eutectic PCM was analyzed by a thermogravimetric analyzer (TGA). The TGA analysis depicts the mass loss (%) with incremental temperature as shown in Figure 8. The decomposition temperatures for caprylic acid and stearyl alcohol were 156.7 °C and 239.8 °C, whereas the decomposition starts at 159.8 °C for the eutectic mixture. However, the decomposition temperature was higher than its melting point temperature, which would not affect its performance during temperature increase due to thermal fluctuation. The TGA analysis shows a wide safe operating temperature range for the prepared eutectic PCM. Therefore, the caprylic acid-stearyl alcohol holds good thermal stability and is safe to utilize for real-time applications.

**Figure 9:**
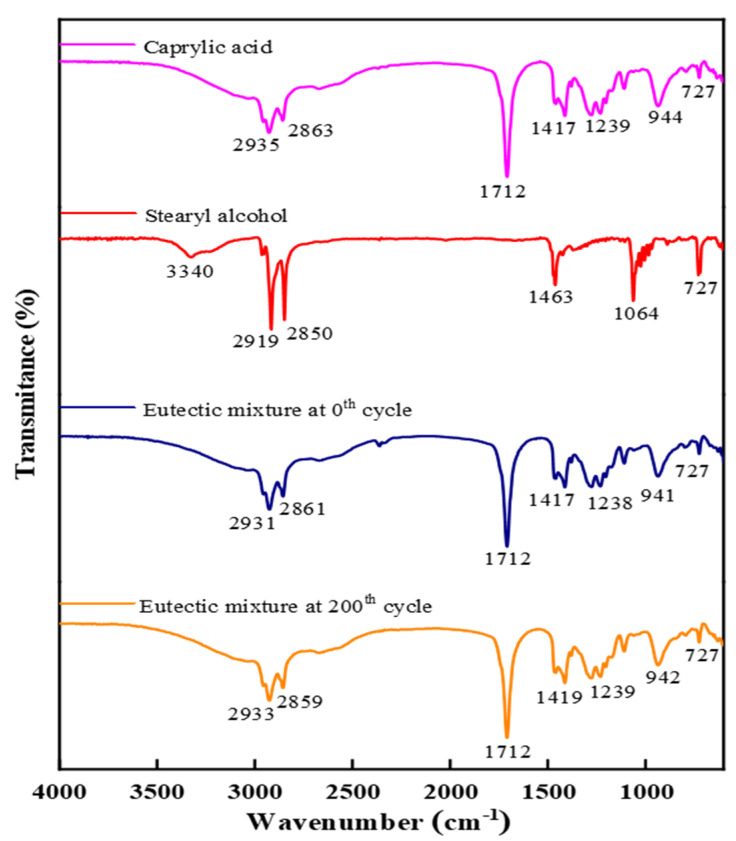
FT-IR of the pure materials and prepared eutectic PCM.

Fourier-transform infrared spectroscopy (FT-IR) gives insight into the chemical structure of the eutectic PCM to indicate its chemical stability. The FT-IR spectral data for caprylic acid, stearyl alcohol, and eutectic PCM at 0 cycles and 200 cycles are shown in Figure 9. The spectral data for the pristine materials were similar to the results reported in previous studies [37,38]. The spectral peaks of caprylic acid at 2935 cm^−1^ and 2863 cm^−1^ indicate O–H and C–H vibration stretching. The spectral peaks at 1712 cm^−1^ and 1417 cm^−1^ represent C=O and –CH_2_ stretching, respectively. The 1239 cm^−1^ and 727 cm^−1^ show the C–O stretching. The absorption peak at 3340 cm^−1^ for stearyl alcohol signifies the –OH stretching vibration. The –CH_2_ is represented by 1463 cm^−1^, and C–O appears by 1064 cm^−1^ and 727 cm^−1^. The FT-IR results for the pure materials and eutectic PCM depict that the materials are chemically stable and the chemical structure is not varied. The prepared eutectic PCM is chemically stable for 200 thermal cycles and no change was evidenced in the chemical structure. The caprylic acid-stearyl alcohol holding good chemical stability makes it a potential candidate for real-time cooling applications.

### 3.2. Accelerated Thermal Cycling of Caprylic Acid-Stearyl Alcohol Binary Mixture

**Figure 10:**
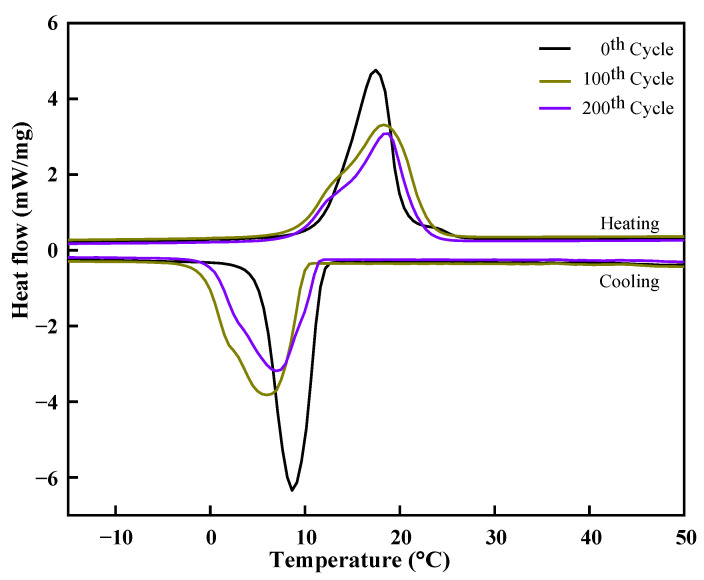
DSC thermographs of the eutectic PCM for 0th, 100th, and 200th thermal cycles.

The thermal properties of the prepared eutectic PCM are essential to be investigated for various melting/freezing thermal cycles in order to analyze its performance for long-term applications. In this study, 200 melting/freezing thermal cycles were employed with a regular interval of 100 cycles for the evaluation of thermal stability. DSC thermographs were attained for the 0th, 100th, and 200th thermal cycles to predict the variation in melting/freezing point temperature and latent heat of fusion and it is shown in Figure 10. As a result, the variation in the onset melting/freezing point temperature and latent heat of fusion after 100 cycles was varied by 2.2 °C/1.9 °C and 4.7 /0.2 J/g, respectively. The change in the thermophysical properties can be quantified with the relative percentage difference (*RPD*), as presented in Equation (2) [19]:

(2) $$RPD=\frac{X_{n,i}-X_{o,i}}{X_{o,i}}\times 100$$

where *X_n,i_* represents the thermal properties for the nth cycle and *X_o,i_* for the referenced cycle. The subscript *i* is the property of eutectic PCM melting/freezing point temperature and latent heat of fusion. The variation in the thermophysical properties for 200 thermal cycles is measured by using Equation (2) and presented in Table 3. Caprylic acid-1-dodecanol eutectic mixture (70:30), withs a melting point temperature of 6.52 °C and a latent heat of fusion of 171.06, was examined for 120 thermal cycles. The result showed the eutectic mixture reported in the literature and prepared eutectic mixture in this work has approximately similar phase change enthalpy difference after 120 thermal cycles [23]. The positive sign shows an increase, whereas a negative sign represents a decrease in the thermal properties compared to the reference values. The maximal variation for phase change temperature and latent heat of fusion was −16.1% and −18.9%, respectively. The reported fatty acid-fatty acid eutectic mixture for cold thermal energy storage has an RPD value of −35–+25% for the latent heat of fusion and a melting temperature difference of −1.69 °C–4.33 °C [39]. Thus, the change in the thermal properties of the prepared eutectic PCM was in good range and made the material a good fit for its utilization in cold thermal energy storage applications.

### 3.3. Corrosion Test of Caprylic Acid-Stearyl Alcohol Binary Mixture

**Figure 11:**
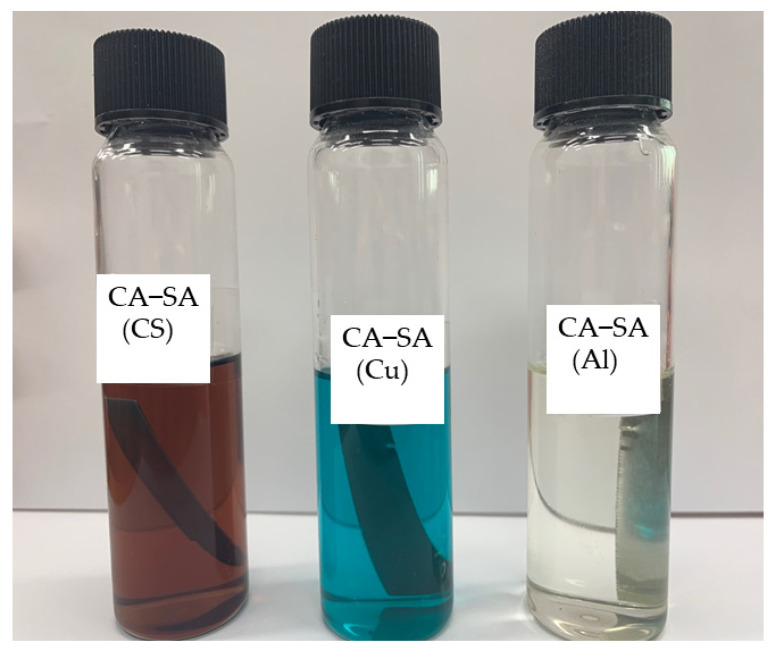
Metal samples in eutectic PCM after 12 weeks.

**Figure 12:**
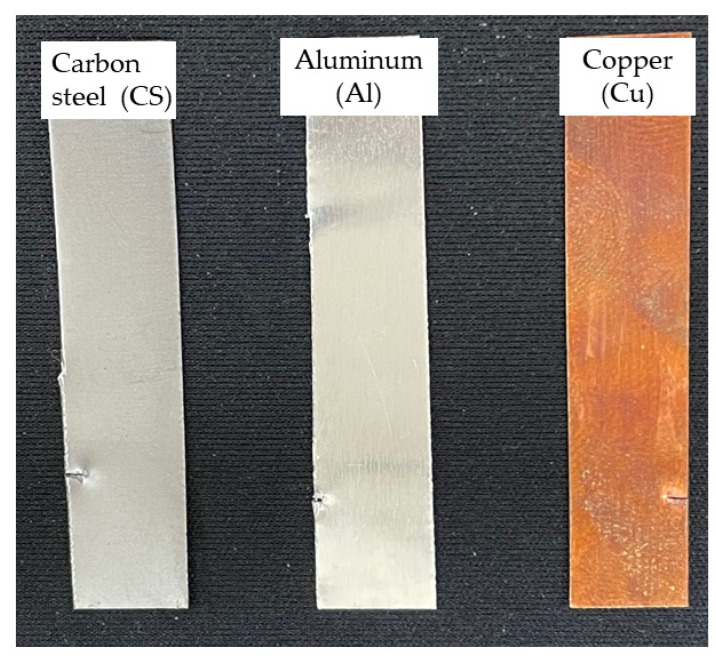
Metal strips after 12 weeks of corrosion testing.

The different metals, namely aluminum, copper, and carbon steel, which are utilized for the manufacturing of thermal energy storage components, were analyzed for compatibility of the prepared eutectic PCM. The metal strips immersed in the caprylic acid-stearyl alcohol (CA-SA) are shown in Figure 11. The color of the solution as observed for the copper and carbon steel changed to bluish-green and dark brown, respectively. The bluish-green color of copper immersion indicates corrosion, which is due to the formation of hydrated metal chloride salts, and the dark brown for carbon steel was from iron oxide formation. The aluminum immersed in the eutectic PCM did not show any color change after 12 weeks of corrosion. Figure 12 shows the metals strip after 12 weeks of corrosion testing. The copper strip shows slight black deposition on its surface, a mild brown layer was seen on the carbon steel surface, and minute white deposition was formed for aluminum, which indicates the corrosion.

**Figure 13:**
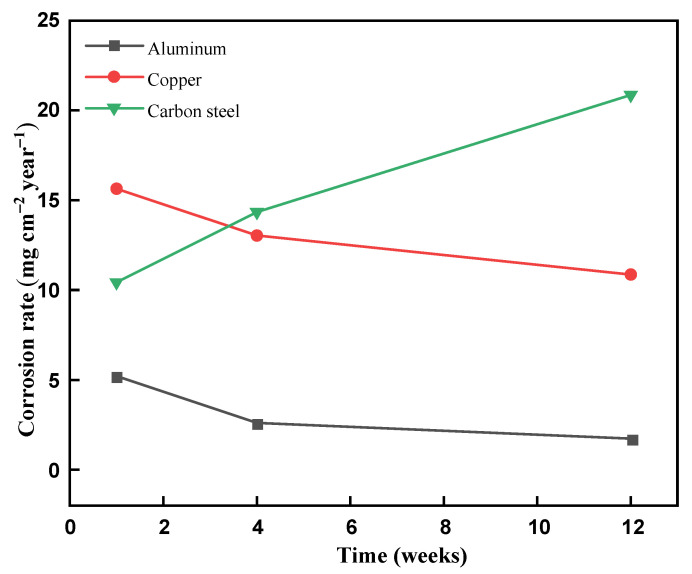
Corrosion rate of aluminum, copper, and carbon steel in the eutectic mixture.

The corrosion rate from the mass loss was measured for 1, 4, and 12 weeks time and it is presented in Figure 13. From Figure 12, it is deduced that the corrosion rate for copper and aluminum was decreased with an increase in the exposure duration of the metal strips in the eutectic PCM. The decrement corrosion rate was due to the formation of an oxidized layer in-between the metal strip and eutectic PCM, which limits the direct contact of the metal strip. For the first week, the corrosion rate for carbon steel was 10.42 mg/cm^2^.year, and it tended to rise as the binary mixture’s acidic nature is exposed to excess iron (Fe) content. A general guide used in the industries for the corrosion rate and weight loss is presented in Table 4 [16]. Following the guidelines, copper and carbon steel can be utilized based on caution for specific applications. However, copper and carbon steel are not recommended for the manufacturing of cold thermal energy storage components. Aluminum is safe due to its low corrosion rate, which can be used for long-term applications and utilized to manufacture cold thermal energy storage systems.

## 4. Conclusions

In this work, a novel eutectic PCM of caprylic acid and stearyl alcohol with the eutectic composition of 90:10 was prepared. The onset melting and freezing temperature for the caprylic acid-stearyl alcohol eutectic mixture was 11.4 °C and 11.8 °C with the latent heat of fusion 154.4 J/g and 150.5 J/g, respectively. Caprylic acid-1-dodecanol eutectic mixture (70:30) with a melting point temperature of 6.52 °C and latent heat of fusion 171.06 J/g was examined for 120 thermal cycles. The result showed the eutectic mixture reported in the literature and prepared eutectic mixture in this work has approximately similar phase change enthalpy difference after 120 thermal cycles [23]. The melting point temperature of the eutectic mixture prepared was in a good range and therefore can be utilized for various cooling applications. Thermogravimetric analysis shows the decomposition temperature was 159.8 °C, which is higher than the melting point temperature and would be safe if the temperature is increased by thermal fluctuation. After 200 thermal cycles, no significant change in the thermal properties and chemical structure of the prepared eutectic PCM was evidenced. The reported thermal conductivities for pure caprylic acid and stearyl alcohol in open literature were 0.15 W/m.K and 0.248 W/m.K, which are in good agreement with the prepared eutectic PCM. The thermal conductivity in the solid and liquid phase is in a suitable range, which would positively impact energy storage/retrieval performance. A corrosion test indicates that aluminum is compatible with the prepared binary mixture and can be utilized for long duration with minimal service. In contrast, copper and carbon steel were not recommended due to the high corrosion rate. Accordingly, it was confirmed that the caprylic acid-stearyl alcohol eutectic PCM was stable, safe for real operating conditions, and compatible with some metal for the cold thermal energy storage system.

## Figures and Tables

**Table 1:** Summary of PCM binary mixture reported for cooling application.

PCM Binary Mixture	Eutectic Composition	Melting Point Temperature (℃)	Latent Heat of Fusion (J/g)	Ref.
Capric acid-cetyl alcohol	70:30	22.89	144.92	[19]
Lauric acid-myristyl alcohol	40:60	22.46	151.5	[20]
Lauryl-cetyl alcohol	80:20	20.01	191.63	[25]
Caprylic acid-1-dodecanol	70:30	6.52	171.06	[21]
Capric acid-dodecanol	60:40	8.9	156.6	[27]
Caprylic-palmitic acid	90:10	11.33	116.47	[26]
Capric-lauric acid	65:35	18	140.8	[22]
Caprylic-nonanoic acid	81.4:18.6	7.6	123	[24]
Caprylic acid-stearyl alcohol	90:10	11.4	154.1	Present study

**Table 2:** Thermo-physical properties of pristine materials.

Materials	IUPAC Name	Melting		Solidification			Molar Mass
		*T_Peak_* (°C)	Δ*H_m_* (J/g)	*T_Peak_* (°C)		Δ*H_f_* (J/g)	g/mol
Caprylic acid	Octanoic acid	18.4	142.6	4.1		154.6	144.21
Stearyl alcohol	1-Octadecanol	61.4	240.7	52.4	48.1	257.9	270.49

**Table 3:** Relative percentage difference of thermophysical properties after thermal cycling.

No. of Cycles	*T_m_* °C	*T_f_* °C	Δ*H_m_* J/g	Δ*H_f_* J/g	RPD of *T_m_* %	RPD of *T_f_* %	RPD of Δ*H_m_* %	RPD of Δ*H_m_* %
0	11.4	11.8	154.4	150.5				
100	9.8	9.9	149.7	150.3	−14.0	−16.1	−3.04	−0.13
200	10.8	11.4	125.2	124.3	−5.2	−3.3	−18.9	−17.4

**Table 4:** Guide for corrosion and weight loss in industries [19].

mg/cm · Year	mm/Year	Recommendation
>1000	2	Completely destroyed within days
100–999	0.1–1.99	Not recommended for service greater than a month
50–99	0.1–0.19	Not recommended for service greater than one year
10–49	0.02–0.09	Caution recommended, based on the specific application
0.3–9.9	-	Recommended for long term service
<0.2	-	Recommended for long term service; no corrosion, other than as a result of surface cleaning was evidenced

## Data Availability

Not applicable.
